# Biflavones from *Platonia insignis* Mart. Flowers Promote In Vitro Antileishmanial and Immunomodulatory Effects against Internalized Amastigote Forms of *Leishmania amazonensis*

**DOI:** 10.3390/pathogens10091166

**Published:** 2021-09-10

**Authors:** Érika Alves Bezerra, Michel Mualém de Moraes Alves, Simone Kelly Rodrigues Lima, Emanuelly Elanny Andrade Pinheiro, Layane Valéria Amorim, José de Sousa Lima Neto, Fernando Aécio de Amorim Carvalho, Antônia Maria das Graças Lopes Citó, Daniel Dias Rufino Arcanjo

**Affiliations:** 1 Department of Biophysics and Physiology, Federal University of Piauí, Teresina 64049-550, Brazil; erikka.ab@gmail.com (É.A.B.); simonelima.nut@gmail.com (S.K.R.L.); 2 Medicinal Plants Research Center, Federal University of Piauí, Teresina 64049-550, Brazil; mualemmichel@ufpi.edu.br (M.M.d.M.A.); layane.valeria@hotmail.com (L.V.A.); famorim@ufpi.edu.br (F.A.d.A.C.); 3 Department of Education, Federal Institute of Maranhão, Bacabal 65080-805, Brazil; 4 Department of Chemistry, Federal University of Piauí, Teresina 64049-550, Brazil; emanuellyeandradep@gmail.com (E.E.A.P.); gracito@ufpi.edu.br (A.M.d.G.L.C.); 5 Faculty of Pharmacy, Federal University of Piauí, Teresina 64049-550, Brazil; limaneto@ufpi.edu.br

**Keywords:** antileishmanial activity, cytotoxicity in macrophages, medicinal plants, immunomodulation, natural products, hydroalcoholic extract, ethyl acetate fraction, biflavone mixture, *Platonia insignis*

## Abstract

Leishmaniasis is an infectious disease that affects millions of people worldwide, making the search essential for more accessible treatments. The species *Platonia insignis* Mart. (Clusiaceae) has been extensively studied and has gained prominence for its pharmacological potential. The objective of this work was to evaluate the antileishmania activity, cytotoxic effect and activation patterns of macrophages of hydroalcoholic extract (EHPi), ethyl acetate fractions (FAcOEt) and morelloflavone/volkensiflavone mixture (MB) from *P. insignis* flowers. EHPi, FAcOEt and MB demonstrated concentration-dependent antileishmania activity, with inhibition of parasite growth in all analyzed concentrations. EHPi exhibited maximum effect at 800 μg/mL, while FAcOEt and MB reduced the growth of the parasite by 94.62% at 800 μg/mL. EHPi, FAcOEt and MB showed low cytotoxic effects for macrophages at 81.78, 159.67 and 134.28 μg/mL, respectively. EHPi (11.25 µg/mL), FAcOEt (11.25 and 22.5 µg/mL) and MB (22.5 µg/mL) characterized the increase in lysosomal activity, suggesting a possible modulating effect. These findings open for the application of flowers from a *P. insignis* flowers and biflavones mixture thereof in the promising treatment of leishmaniasis.

## 1. Introduction

Neglected tropical diseases (NTDs), prevalent in tropical and subtropical regions, affect more than one billion people worldwide and mainly affect populations living in poorer regions where hygiene, food and sanitation conditions are more precarious and where they are exposed to infectious vectors and domestic animals [1]. Leishmaniasis continues to be a major health problem of the world. There are four main forms of the disease: visceral leishmaniasis, post-kala-azar dermal leishmaniasis, cutaneous leishmaniasis and mucocutaneous leishmaniasis. Visceral leishmamiasis is the most serious and is almost always fatal untreated, while cutaneous is the most common [2]. 

Leishmaniasis is a complex of infectious parasitic diseases caused by protozoa of the genus *Leishmania*, which are transmitted by the bite of infected female phlebotomine sandflies [1]. Among them, leishmaniasis is estimated to be second in mortality and fourth in morbidity. An estimated 700,000 to 1 million new cases occur annually. [1,3]. The drug of first choice for the treatment of leishmaniasis is N-methyl glucamine antimoniate, known commercially as Glucantime®, and Amphotericin B is the second choice [4,5]. However, the currently available treatment requires continuous clinical and laboratory monitoring, exhibits severe side effects, leads to intolerance, sometimes causes treatment dropout, and is expensive [6]. In this context, it is essential to search for new treatments that have low toxicity and lower cost and are easily accessible.

Medicinal plants have become an alternative in the search for substances with antileishmanial activity because they are promising sources for the synthesis of a wide variety of new drugs due to the magnitude of their biodiversity and the amount of biologically active chemical compounds they contain [7,8].

*Platonia insignis* Mart. (Clusiaceae family) is a promising species in the search for bioactive compounds. It is widely used in the treatment of skin diseases, spider and snake bites, rheumatic arthritis and is also used as a cicatrizing agent [9]. Trials performed with the seeds of the fruits have demonstrated wound-healing and anti-inflammatory activities [10], anticonvulsant [11], antileishmania [12], immunomodulatory [13], antimicrobial and anti-inflammatory activities [10]. Furthermore, the fruit pulp has an antioxidant activity [14], and the fruit peels have been used for the treatment of eczema and arthritis [15].

In our group, Silva et al. [16] isolated and identified a mixture of two biflavonoids (moreloflavone and volkensiflavone) and a polyisoprenylated benzophenone garcinielliptona FC (GFC) from the hydroalcoholic extract of *P. insignis* flowers. These compounds have proven to be responsible for important pharmacological activities attributed to the species. Anti-HIV activity [17], antibacterial [18], anti-inflammatory [19], antioxidant [20] and antiparasitic activities [21] were associated with morelloflavone, while volkensiflavone was reported to have analgesic, antibacterial and antitumor activities [20]. 

In view of the above, the components of *P. insignis* deserve to be highlighted as a potential novel source of compounds for the chemotherapy of leishmaniasis, and for which treatment is a critical problem.

## 2. Results

### 2.1. Effect of EHPi, FAcOet and MB on Promastigotes of L. amazonensis

EHPi, FAcOEt and MB demonstrated concentration-dependent antileishmanial activity with inhibition of parasite growth at all concentrations analyzed. EHPi exhibited maximum effect at 800 μg/mL, while FAcOEt and MB reduced parasite growth by 94.62% at 800 μg/mL. The 50% inhibitory concentration (IC_50_) values obtained from the assay on promastigotes are shown in Table 1. 

### 2.2. Cytotoxicity and Hemolysis Assay

**Figure 1:**
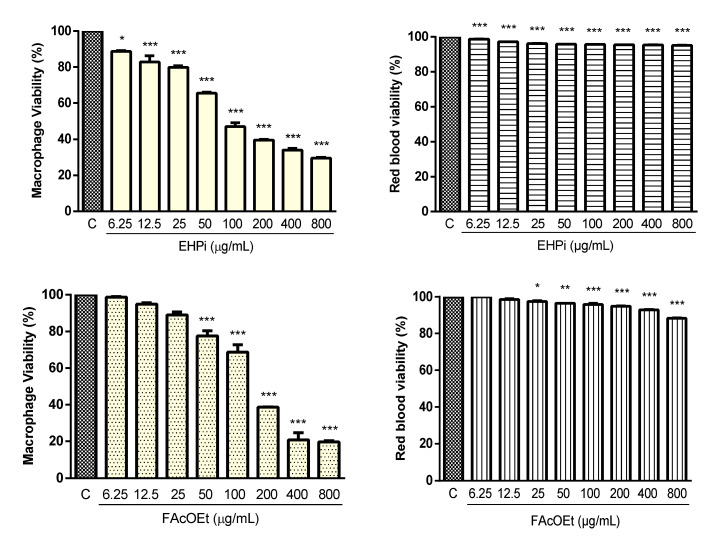

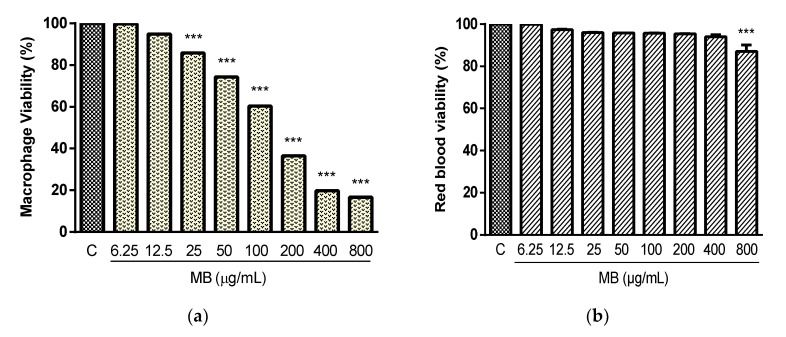
Cytotoxic effects of EHPi, FAcOEt and MB against BALB/c murine peritoneal macrophages (**a**) and sheep red blood cells (**b**). Macrophages and red blood cells were incubated with GFC for 48 h. Macrophage viability was evaluated using tetrazolium salt (MTT) test. Data are presented as mean ± SEM of three experiments performed in triplicate. * *p* < 0.05; ** *p* < 0.01; *** *p* < 0.001 when compared with control (C) or Amph B. One-way ANOVA followed by Bonferroni’s post hoc test.

EHPi, FAcOEt and MB showed low cytotoxic effects for murine macrophages and sheep erythrocytes (Figure 1). There was a statistically significant reduction in macrophage viability by MTT assay from the lowest concentration tested (6.25 μg/mL) for EHPi, while for FAcOEt and MB, the reduction occurred from 50 and 25 μg/mL, respectively, with CC50 of 81.78 μg/mL for EHPi, 159.67 μg/mL for FAcOEt and 134.28 μg/mL for MB. Amphotericin B, conversely, a drug that shows high toxicity against murine macrophages, showed CC50 of 8.75 μg/mL (Table 1). At the maximum concentration tested (800 μg/mL), EHPi reduced RBC viability by 4.81%, while FAcOEt and MB reduced it by 11.74% and 12.86%, respectively. 

### 2.3. Effects of EHPi, FAcOEt and MB on L. amazonensis Infection of Macrophages

**Figure 2:**
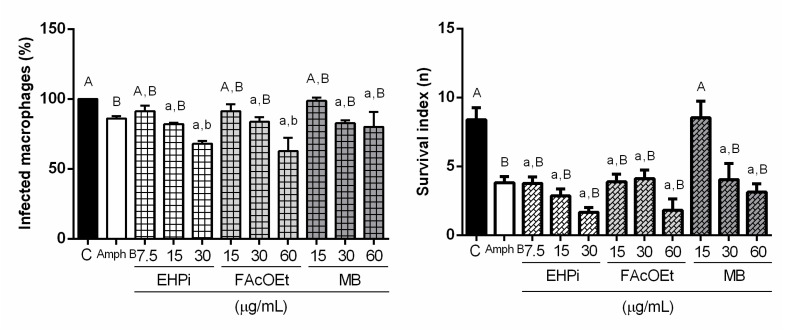
Effects of EHPi, FAcOEt and MB and amphotericin B on infected macrophages and survival index of BALB/c murine macrophages infected with *L. amazonensis*. Cells were treated with GFC or Amph B for 48 h. Data are presented as mean ± SEM of three experiments performed in triplicate. “A” and “B” when it does not differ statistically from the control (C) or amphotericin B (Amph B), respectively; “a” and “b” when differences between control (C) or Amph B, respectively, are significant. One-way ANOVA followed by Bonferroni’s post hoc test.

**Figure 3:**
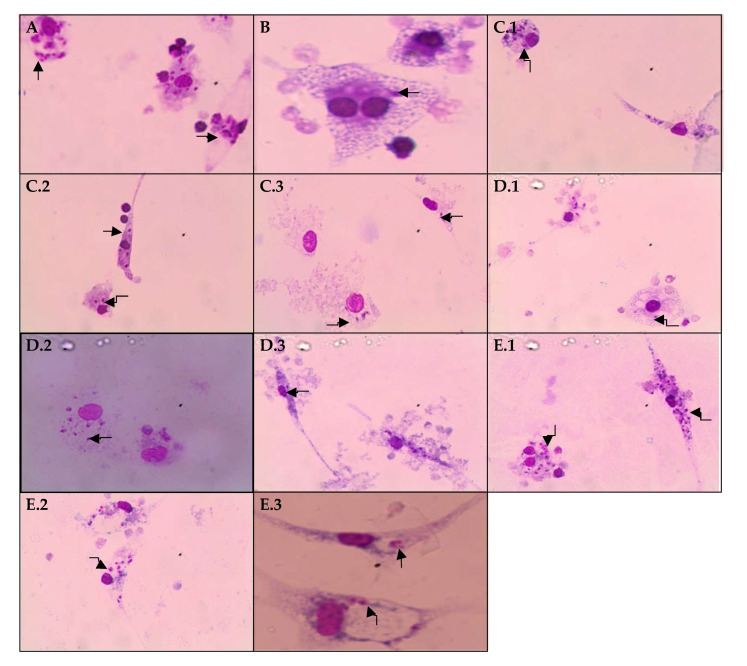
Macrophages experimentally infected by *Leishmania amazonensis* (**A**). Amph B was used as a positive control at the concentration of 0.2 μg/mL (**B**). For EHPi treatment, concentrations of 7.5 (**C.1**), 15 (**C.2**), and 30 μg/mL (**C.3**) were used, for FAcOEt treatment, concentrations of 15 (**D.1**), 30 (**D.2**) and 60 μg/mL (**D.3**) and for MB treatment, concentrations of 15 (**E.1**), 30 (**E.2**) and 60 μg/mL (**E.3**). The arrows indicate macrophage-internalized amastigote forms of *L. amazonensis*.

EHPi at concentrations of 7.5 and 15 μg/mL and MB at concentrations of 30 and 60 μg/mL reduced the number of infected macrophages compared to control, but when compared to amphotericin B, there were no significant reductions. FAcOEt at 15 and 30 μg/mL and MB at 15 μg/mL showed no action on reducing infection. However, EHPi at 30 μg/mL and FAcOEt at 60 μg/mL reduced infection by 68% and 62.66% respectively, while amphotericin B decreased the number of infected macrophages to approximately 86% of the concentration of 0.2 μg/mL (Figure 2 and Figure 3). 

### 2.4. Determination of Lysosomal Activity and Phagocytic Capacity

**Figure 4:**
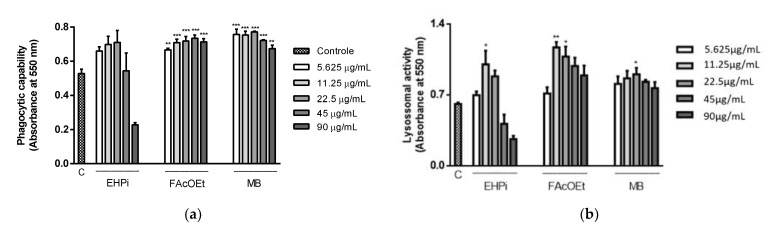
Effects of EHPi, FAcOEt and MB on lysosomal activity (**a**) and phagocytic capability (**b**). Murine peritoneal macrophages were treated at ranging concentrations for 48 h. Lysosomal activity and phagocytic capacity were assessed by quantification of neutral red (NR). Phagocytic capability was assessed by the incorporation of zymosan to NR, solubilized by the extraction solution. Data are presented as mean ± SEM of three experiments performed in triplicate. * *p* < 0.05; ** *p* < 0.01; *** *p* < 0.001 when compared with control (C). One-way ANOVA followed by Bonferroni’s post hoc test.

To evaluate the activation mechanisms of macrophages induced by EHPi, FAcOEt and MB, the capacity of these cells, when activated, to retain neutral red and zymosan particles, was analyzed by incubating the test samples (EHPi, FAcOEt and MB) with macrophages infected with *L. amazonensis*. EHPi promoted a statistically significant increase in lysosomal volume at the concentration of 11.25 μg/mL, and at higher concentrations, there begins to be a concentration-dependent reduction in activation (Figure 4a). EHPi did not induce an increase in the phagocytic capacity of macrophages for any of the concentrations tested (5.625 to 90 μg/mL) (Figure 4b). FAcOEt promoted increased lysosomal volume at concentrations of 11.25 and 22.5 μg/mL, and although at concentrations of 45 and 90 μg/mL there was a reduction in activation, its volume remained larger than that of the control. MB induced increased lysosomal volume at the concentration of 22.5 μg/mL. FAcOEt and MB induced a statistically significant increase in the phagocytic capacity of macrophages at all concentrations analyzed (5.625 to 90 μg/mL).

### 2.5. Determination of Nitrite Production

**Figure 5:**
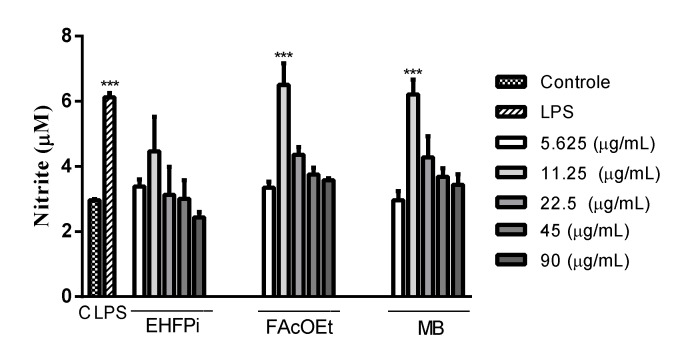
Nitrite measurement in infected or non-infected BALB/c murine peritoneal macrophages treated with EHPi, FAcOEt and MB for 24 h. The culture supernatant was mixed in equal parts with the Griess reagent. LPS (lipopolysaccharide from *Escherichia coli*; 2 μg/mL) was used as positive control. Data are presented as mean ± SEM of three experiments performed in triplicate. *** *p* < 0.05 when compared with control group *** *p* < 0.05 when compared with LPS group. One-way ANOVA followed by Bonferroni’s post hoc test.

The evaluation of nitric oxide induction by macrophages is given by quantifying the concentration of nitrite present in the medium. EHPi did not induce NO production at any of the concentrations analyzed (5.625 to 90 μg/mL), whereas FAcOEt and MB significantly increased NO production at the concentration of 11.25 μg/mL (Figure 5).

## 3. Discussion

Medicinal plants have shown to be a promising alternative for the treatment of various diseases, including leishmaniasis [22,23]. The drugs currently available for the treatment of this pathology are limited and present high risks of toxicity for patients [24]. Among the great diversity of the Brazilian flora, *Platonia insignis,* belonging to the Clusiaceae family, deserves to be highlighted.

Assays performed with the seeds of *Platonia* fruits demonstrated potential antileishmanial activity, with IC_50_ of 25.78 μg/mL, for Garcinielliptone FC (GFC) on promastigote forms of *L. amazonensis* [12]. The ethanolic extract, fukugetin and morelloflavone obtained from the fruits of *Garcinia brasiliensis*, also belonging to the Clusiaceae family, showed antileishmanial action on *L. amazonensis* promastigotes forms, with IC_50_ of 15, 3.2, and 0.1390 μM, respectively. Morelloflavone showed IC_50_ 0.2900 μM on amastigotes. The samples, EHPi, FAcOEt and MB promoted inhibition of the growth of *L. amazonensis* promastigotes at all concentrations analyzed, with IC_50_ of 30.80, 23.04 and 45.71 μg/mL, but with antileishmanial action at concentrations considerably higher than that of amphotericin B.

The use of medicinal plants for the prevention, alleviation of symptoms and cure of diseases is a widespread practice worldwide [25]. However, they are widely used indiscriminately, which can cause cytotoxic and genotoxic alterations that lead to the development of other pathologies [26]. It is important that studies that analyze the action of natural products at the cellular level be conducted in order to ensure the safe use by the population as well as to support further studies [27]. Cytotoxicity, MTT and hemolytic activity assays, performed with EHPi, FAcOEt and MB showed low cytotoxicity for macrophages, essential cells in the defense mechanisms of leishmaniasis, and for sheep erythrocytes, with CH50 being at concentrations well above 800 μg/mL, indicating greater selectivity for the parasite than for mammalian cells. Cytotoxicity assays with the hexanic extract of bacuri fruit seeds showed similar results, with a CH50 of 90.03 µg/mL for murine macrophages, with a confidence interval between 70.72 and 114.6 µg/mL and 3% hemolysis in human erythrocytes at the concentration of 400 µg/mL [13], reaffirming its use as safe.

Immunostimulation is essential and effective in the prevention and treatment of diseases that compromise the immune system. Medicinal plants have become a promising alternative in the development of drugs for this purpose, proving to be adequate in the prevention and treatment of infections [28]. The existing dichotomy between Th1 and Th2 immune cell response in patients affected by leishmaniasis has led to the investigation of therapeutic alternatives that direct immunomodulatory effect to the Th1 response and not only target the parasite [29]. Macrophages play a central role in modulating the immune response, and several systems are activated in order to fight an infection. It is possible to evaluate the ability that many chemical compounds have to activate these cells through in vitro assays [30,31]. EHPi (11.25 µg/mL), FAcOEt (11.25 and 22.5 µg/mL) and MB (22.5 µg/mL) retained neutral red particles, characterizing increased lysosomal activity. Increased lysosomal activities, thus, suggest a possible defense of these cells. These data are in agreement with the study conducted by Lustosa et al. [13], in which the hexanic extract (EHSB), produced from the seeds of the fruits, promoted an increase in lysosomal volume at concentrations of 12.5, 25 and 50 µg/mL. The activation of macrophages by using zymosan particles stained with neutral red was another parameter evaluated. Zymosan stimulates defense cells to induce Th1-type response, leading to an increase in interferon gamma (IFN) production and phagocytic capacity [32]. Although the extract did not activate this mechanism, FAcOEt and MB (5.625 to 90 μg/mL) induced statistically significant increases in the phagocytic capacity of macrophages. Lustosa et al. [13] demonstrated that EHSB induced increased phagocytic capacity of macrophages by 42.9%, 49.8%, 53.7% and 71.2% at concentrations of 25, 12.5, 6.25 and 3.12 μg/mL, respectively. The cytotoxic effect against pathogenic microorganisms is indirectly realized by nitric oxide (NO) produced by macrophages in the processes of inflammation, angiogenesis and defense mechanisms against pathogenic microorganisms [33,34]. Its synthesis is considered one of the most important defense mechanisms, as the parasite is able to inhibit the expression or activity of the inducible nitric oxide synthase (iNOS) enzyme to survive inside macrophages [35]. FAcOEt (11.25 μg/mL) and MB (11.25 μg/mL) promoted increased NO production, and reduced activation of these mechanisms was observed at higher concentrations. Possibly, the higher concentrations tested lead to reduced cell viability. In the study conducted by Lustosa et al. [13], the hexanic extract induced NO production at all concentrations analyzed (3.12 to 100 μg/mL). The mechanisms of antiparasitic action of flavonoids are not yet evidenced, but it is known that they exert an effect on the generation of reactive oxygen species [36] and increase the production of pro-inflammatory cytokines, such as IFN [37]. 

BALB/c murine peritoneal macrophages were infected with promastigotes of *L. amazonensis* and incubated with EHPi, FAcOEt and MB to evaluate infection and infectivity compared with control. EHPi (30 μg/mL) and FAcOEt (60 μg/mL) statistically reduced macrophage infection but were not able to reduce infectivity when compared to positive control (amphotericin B). 

The findings suggest that EHPi, FAcOEt, and MB may possibly be used as a supportive therapy to conventional antileishmania chemotherapy, as they have been shown to be immunostimulatory proponents that would aid in the fight against the infection. Furthermore, additional studies are needed to elucidate the immunological mechanisms involved in these effects.

## 4. Materials and Methods

### 4.1. Samples

The samples used to perform the biological assays, hydroalcoholic extract (EHPi), ethyl acetate fraction (FAcOEt) and biflavonoids mixture (MB) were obtained by developing the work of Silva et al. [16], performed in the Laboratory of Organic Geochemistry of the Federal University of Piauí (LAGO/UFPI). The flowers of *Platonia insignis* Mart. were collected in the municipality of Parnarama, Maranhão state, in August 2014. The exsicata is deposited in the Graziella Barroso Herbarium, of the Federal University of Piauí (UFPI), under the ICN number TEPB 27174. The petals of fresh flowers of *P. insignis* were ground and macerated in a solvent mixture EtOH/H_2_O, in the ratio of 7:3 (*m*/*v*), in the ratio of 1:5 plant material/solvent (*m*/*v*) at room temperature, for 9 days. Subsequently, the hydroalcoholic extract (EHPi) was rotaevaporated and lyophilized. The EHPi was resuspended in MeOH/H_2_O 9:1 (*v*/*v*) and subjected to liquid-liquid partitioning with solvents in increasing order of polarity. The ethyl acetate fraction, FAcOEt, was obtained with a mass of 3.82 g and η = 26.56%. From mass spectrometry with electrospray ionization in negative mode coupled to mass spectrometer (ion trap hybrid) (ESI-IT-MS) in scan mode, a mixture of biflavonoids, morelloflavone and volkensiflavone, stood out as majorities in the hydroalcoholic extract. To identify the constituents present in the FAcOEt fraction, silica dry column chromatography was performed, obtaining a subfraction, eluted with MeOH/H_2_O 1: 1 (*v*/*v*), presenting as a yellow amorphous solid. After ESI-MS analysis, in the negative ionization mode, and NMR, the morelloflavone compounds were identified, with the majority molecular form C_30_H_19_O_11_, and the volkensiflavone, whose molecular form is C3_0_H_19_O_10_. 

### 4.2. Sample Preparation

The hydroalcoholic extract (EHPi), the ethyl acetate fraction (FAcOEt) and the biflavone mixture (MB) were diluted in dimethyl sulfoxide (DMSO) to a concentration of 80 mg/mL to perform the assays. To obtain the desired concentrations (6.25 to 800 µg/mL) for each protocol, the solutions were diluted in RPMI 1640 medium for macrophages or Schneider’s medium for parasites, without exceeding 0.2% DMSO.

### 4.3. Animals

For the study, 4 BALB/c mice (20 and 30 g, males and females, aged between 4 and 6 weeks) were used. They were kept on a 12 hr light/dark cycle, with free access to water and food. The animals were anesthetized and euthanized with overdose of anesthetics: thiopental (150 mg/kg) and lidocaine (10 mg/kg) via intraperitoneal and subsequently euthanized by cervical dislocation, according to resolution no. 1000/2012 of the Federal Council of Veterinary Medicine, Brazil. All experiments in this study were approved by the Animal Research Ethics Committee of UFPI (n^o^. 321/17).

### 4.4. Parasites and Cells

Promastigotes of *Leishmania *(leishmania) *amazonensis* (IFLA/BR/67/PH8) were obtained from the Núcleo de Pesquisas em Plantas Medicinais da Universidade Federal do Piauí to perform the antileishmania activity assays. The parasites were grown in Schneider’s medium (Sigma-Aldrich, Saint-Louis, MO, USA), supplemented with 10% fetal bovine serum (FBS) (Sigma, USA), 100 U/mL penicillin and 100 μg/mL streptomycin (Sigma) and incubated at 26 °C in a Biochemical Oxygen Demand (B. O. D.) [38,39]. Murine macrophages were obtained from the peritoneal cavity of BALB/c mice and cultured in RPMI 1640 medium (Sigma), supplemented with 10% FBS, 100 U/mL penicillin and 100 µg/mL streptomycin at 26 °C).

### 4.5. Antileishmanial Activity on Promastigotes

Promastigotes of *L. amazonensis* in logarithmic growth phase (1 × 10^6^ leishmania/100 µL medium) were seeded into 96-well cell culture plates containing supplemented Schneider’s medium. Vehicle and serial dilutions of EHPi, FAcOEt, and MB samples (800, 400, 200, 100, 50, 25, 12.5, and 6.25 µg/mL) were made on the plates and incubated in a B. O. D. at 26 °C for 48 h. With 6 h remaining, 20 µL of resazurin (1 × 10^−3^ mol/L) was added. Subsequently, the absorbances were read in a Biotek microplate reader (ELx800) at 550 nm. The results were expressed in terms of growth inhibition (%). The negative control was performed with 0.2% DMSO in Schneider’s medium (vehicle) containing 1 × 10^6^ promastigotes and considering 100% parasite viability. The positive control was performed with amphotericin B diluted in Schneider’s medium [22]. Statistical significance was determined using one-way ANOVA followed by Bonferroni’s post hoc test. The whole experiment was performed in triplicate of three isolated experiments. 

### 4.6. Determination of Cytotoxicity

#### 4.6.1. Cytotoxicity on Murine Macrophages

Cytotoxicity analyses on murine macrophages were performed in 96-well plates using the MTT (3-[4,5-dimethylthiazol-2-yl]-2,5-diphenyltetrazolium bromide) colorimetric method [40,41]. Macrophages (2 × 10^5^ per well) were incubated in 100 µL of RPMI 1640 medium at 37 °C in 5% CO_2_ for 4 h for cell adhesion to the coverslips. The wells were washed with RPMI 1640 medium to remove cells that did not adhere. Vehicle, EHPi, FAcOEt and MB were diluted at different concentrations (6.25, 12.5, 25, 50, 100, 200, 400 and 800 µg/mL) were added to the wells and incubated at 37 °C at 5% CO_2_ for 48 h. Subsequently, 10 µL of MTT (5 mg/mL) diluted in 100 µL of RPMI medium was added and incubated at 37 °C in 5% CO_2_ for 4 h. After the incubation period, the supernatant was discarded, 100 μL of DMSO was added, the plates were shaken on a Kleine plate shaker for 30 min, and readings were taken on a Biotek plate reader (Elx800) at 550 nm. The absorbance obtained from the untreated cells was considered as 100% cell viability. Statistical significance was determined using One-way ANOVA followed by Bonferroni’s post hoc test. The whole experiment was performed in triplicate of three isolated experiments. 

#### 4.6.2. Hemolytic Activity

For evaluation of hemolytic activity, as described by Alves et al. [22], sheep erythrocytes were diluted in 80 μL of phosphate-buffered saline (PBS) to adjust concentration to 5% hematocrit and incubated at 37 °C with serial concentrations of vehicle, EHPi, FAcOEt, and MB (6.25 to 800 μg/μL) diluted in 20 μL of PBS. After 1 hr, 200 μL of PBS was added to stop the reaction and the suspensions were centrifuged at 1000×g for 10 min. The supernatants were transferred to 96-well plates. Cell lysis was determined by reading the absorbance at 550 nm. The presence of 100% hemolysis (positive control) and the absence of hemolysis (negative control) were determined by replacing the tested samples with equal volume of sterile Milli-Q water and PBS, respectively. The percentage of hemolysis obtained was determined by comparison with the positive control, 100% hemolysis [42]. Statistical significance was determined using One-way ANOVA followed by Bonferroni’s post hoc test. The entire experiment was performed in triplicate of three isolated experiments.

#### 4.6.3. Activity of EHPI, FAcOEt and MB on *L. amazonensis*-Infected Macrophages

Macrophages (2 × 10^5^ cells/mL) were plated in 24-well plates in supplemented RPMI medium (10% FBS, penicillin 100 U/mL, and streptomycin 100 µg/mL) and incubated at 37 °C in 5% CO_2_ for 3 h for cell adhesion. The macrophages were again incubated with medium containing the promastigote forms (2 × 10^6^), in stationary growth phase, at the ratio of 10 promastigotes to 1 macrophage, in 5% CO_2_ at 37 °C for 4 h. After this time, the supernatants were aspirated to remove the non-internalized parasites. After infection, the wells were washed with 0.1 M PBS. The infected macrophages were incubated with vehicle, EHPi at 7.5, 15 and 30 µg/mL (concentrations non-toxic to cells), FAcOEt and MB at 15, 30 and 60 µg/mL (concentrations non-toxic to cells) and Amphotericin B at 0.2 µg/mL for 48 hrs. Subsequently, the coverslips were removed and fixed with Panotic (Laborclin, Paraná, Brazil). The number of infected macrophages and the number of amastigotes per macrophage were determined by reading the number of parasites in 100 macrophages. Then, selectivity indices were determined by the average ratio of 50% cytotoxic concentration (CC50) for macrophages by 50% effective concentration (EC50) for amastigotes internalized in macrophages [43]. Statistical significance was determined using one-way ANOVA followed by Bonferroni’s post hoc test. The entire experiment was performed in triplicate of three isolated experiments.

### 4.7. Immunomodulatory Mechanisms of Macrophage Activation Induced by EHPi, FAcOEt and MB Samples

#### 4.7.1. Lysosomal Activity

Murine macrophages (2 × 10^5^ cells/well) were incubated with vehicle, EHPi, FAcOEt and MB (5.625 to 90 μg/mL) and with Amphotericin B (0.2 µg/mL) in 96-well plates at 37 °C and 5% CO_2_. After 48 h, 10 µL of the 2% neutral red solution in DMSO was added and incubated for another 30 min. Subsequently, the supernatants were discarded, the wells were washed with 0.9% saline at 37 °C and the neutral red retained in the lysosomal vesicles were solubilized by adding 100 µL of extraction solution (1% glacial acetic acid (*v*/*v*) and 50% ethanol (*v*/*v*) dissolved in distilled water). After 30 min of shaking the plates, the absorbances were read on a Biotek plate reader (Elx800) at 550 nm [44]. Statistical significance was determined using one-way ANOVA followed by Bonferroni’s post hoc test.

#### 4.7.2. Evaluation of Phagocytic Capacity

Murine macrophages (2 × 10^5^ cells/well) were incubated with vehicle, EHPi, FAcOEt and MB (5.625 to 90 μg/mL) and with Amphotericin B (0.2 µg/mL) in 96-well plates at 37 °C and 5% CO_2_. After 48 h, 10 µL of zymosan solution stained with neutral red was added and incubated for 30 min. Subsequently, the plates were washed with 0.9% saline and 100 µL of extraction solution was added. After solubilization in a plate shaker, absorbances were read in a Biotek plate reader (Elx800) at 550 nm [45]. Statistical significance was determined using one-way ANOVA followed by Bonferroni’s post hoc test.

#### 4.7.3. Evaluation of Nitric Oxide Production

To evaluate nitrite production by peritoneal macrophages from BALB/c mice, macrophages were plated (2 × 10^5^ cells/well) in 96-well plates and incubated for 4 h at 37 °C in 5% CO_2_ for cell adhesion. After this time, the supernatants were removed to eliminate macrophages that did not adhere and incubated after the addition of vehicle, EHPi, FAcOEt, and MB at concentrations of 5.625 to 90 μg/mL. After incubation for 24 h at 37 °C in 5% CO_2_, the supernatants were transferred to other 96-well plates for nitrite dosage. Standard curves were prepared with sodium nitrite in RPMI medium in concentration range ranging from 1.1 to 600 μM diluted in RPMI medium. The samples (supernatants) or the solutions prepared to obtain the standard curve with the same volume of Griess reagent (1% Sulfanilamide in H_3_PO_4_ 10% *v*/*v* in Milli-Q® water) were mixed in a 1:1 ratio with 0.1% naphthylenediamine in Milli-Q water and absorbance readings were taken in a Biotek plate reader (ELx800) at 550 nm. DMSO (0.5%) in RPMI medium was used as control [46]. Statistical significance was determined using one-way ANOVA followed by Bonferroni’s post hoc test.

## Figures and Tables

**Table 1:** Antileishmania activity and cytotoxic effects against mammalian cells for EHPi, FAcOEt and MB and amphotericin B (Amph B).

Compounds	Macrophages CC50 (μg/mL)	Red Cells CH50 (μg/mL)	Promastigotes EC50 (μg/mL)	Intramacrophage Amastigotes EC50 (μg/mL) SI_m_	
EHPi	81.78	NT	30.05	6.08	38.55
FacOEt	159.67	NT	23.05	13.44	3.55
MB	134,28	NT	45.71	16.33	0.20
Amph B	8.75	NT	1.74	9.81	43.75

SI, selectivity index; SI_m_, selectivity index intramacrophage amastigotes; NT, non-toxic at tested concentrations; EHPi, hydroalcoholic extract; FacOEt, ethyl acetate fractions; MB, morelloflavone/volkensiflavone mixture; Amph B, amphotericin B.

## Data Availability

Original data from experiments will be available under request.
